# Recurrent lymphangioleiomyomatosis in a lung allograft with COVID-19: autopsy case report and literature review

**DOI:** 10.1186/s42047-021-00095-2

**Published:** 2021-08-28

**Authors:** Sakda Sathirareuangchai, Jenny L. Weon, Suzanne Tintle, Kiran Batra, Shirley X. Yan, Jose R. Torrealba

**Affiliations:** 1 Department of Pathology, University of Texas Southwestern Medical Center, Professional Office Building I Room HP3.392, 5959 Harry Hines Blvd., Dallas, TX 75390 USA; 2 Department of Radiology, University of Texas Southwestern Medical Center, Dallas, TX 75390 USA

**Keywords:** Lymphangioleiomyomatosis, Lung transplantation, Cystic lung disease, COVID-19, Autopsy

## Abstract

Lymphangioleiomyomatosis (LAM) is a rare neoplastic disease of the lung with a characteristic feature of diffuse cystic changes in bilateral lungs. Lung transplantation is considered to be one of the effective treatments in end stage disease. Patients with LAM who underwent lung transplant tend to have more favorable outcome compared to other end stage lung diseases. We report a case of a female patient who was diagnosed with LAM and received bilateral lung transplantation at 45 years of age. Subsequent allograft biopsies were significant for mild acute cellular rejection (Grade A2), for which the immunosuppressive regimen was adjusted accordingly. At 7 years post-transplant, she presented with shortness of breath, cough, and fatigue, and diagnosed with a viral infection. Her chest imaging was unremarkable. However, a transbronchial biopsy was performed to rule out rejection and revealed foci of spindle cells proliferation, with positive HMB-45 and smooth muscle actin immunohistochemical studies, confirming the diagnosis of recurrent LAM. After she was discharged, she was re-admitted 1 week later with severe COVID-19. Her clinical course was complicated by acute respiratory distress syndrome, respiratory failure, and gastrointestinal hemorrhage. The patient passed away on day 36 of hospital stay. Autopsy was requested and confirmed the pathology of recurrent LAM and diffuse alveolar damage from COVID-19.

## Introduction

Lymphangioleiomyomatosis (LAM) is a rare neoplastic disease of the lung, characterized by cystic changes in bilateral lungs from destructive proliferation of the LAM cells. The histomorphology for LAM is notable for the presence of smooth muscle-resembling neoplastic spindle cells, which have a peculiar immunophenotype of both melanocytic and smooth muscle differentiation. LAM is almost exclusively found in middle age females. It also has a strong association with the genetic syndrome of tuberous sclerosis. LAM can be considered as a systemic disease, which multiple organs are involved by a number of PEComatous tumor family, e.g., renal angiomyolipoma. Thus, a detection of driver mutation in cell-free circulating DNA (cfDNA) has been proposed as a potential diagnostic tool in LAM (Zhang et al. 2020).

The clinical manifestation in LAM patient is typical for multiple episodes of pneumothoraces, which happens to be the first presentation in approximately 40% of the patients (Johnson et al. 2010). Other symptoms include dyspnea, chylous pleural effusion and ascites, chest pain, cough, and hemoptysis (Abbott et al. 2005).

Sirolimus (Rapamune®) is an immunosuppressive agent and a first line treatment in symptomatic LAM patient. It exerts the antiproliferative effect by inhibiting mTOR signaling pathway. Eventually, patients with LAM will have declining lung function to the point of end stage respiratory failure. Lung transplant might be a consideration, and many times the only option, in these group of patients.

Herein, we report a case of a middle age female patient who was diagnosed with LAM at the age of 44 years old and underwent bilateral lungs transplant shortly thereafter. She was found to have recurrent LAM in the lung allograft 7 years after the transplant.

## Case presentation

The patient is a 52-year-old female, who first presented to an outside institution with first episode of pneumothorax. She continued to have multiple episodes of pneumothoraces and finally had a transbronchial biopsy at the same outside institution 1 year later. The biopsy revealed interstitial thickening with multiple nodular foci of spindled-appearing cells. Immunohistochemical studies demonstrated patchy positivity in spindled cells for both HMB-45 and smooth muscle actin (SMA), consistent with LAM. She subsequently underwent mechanical pleurodesis for the treatment of recurrent pneumothoraces.

**Fig. 1:**
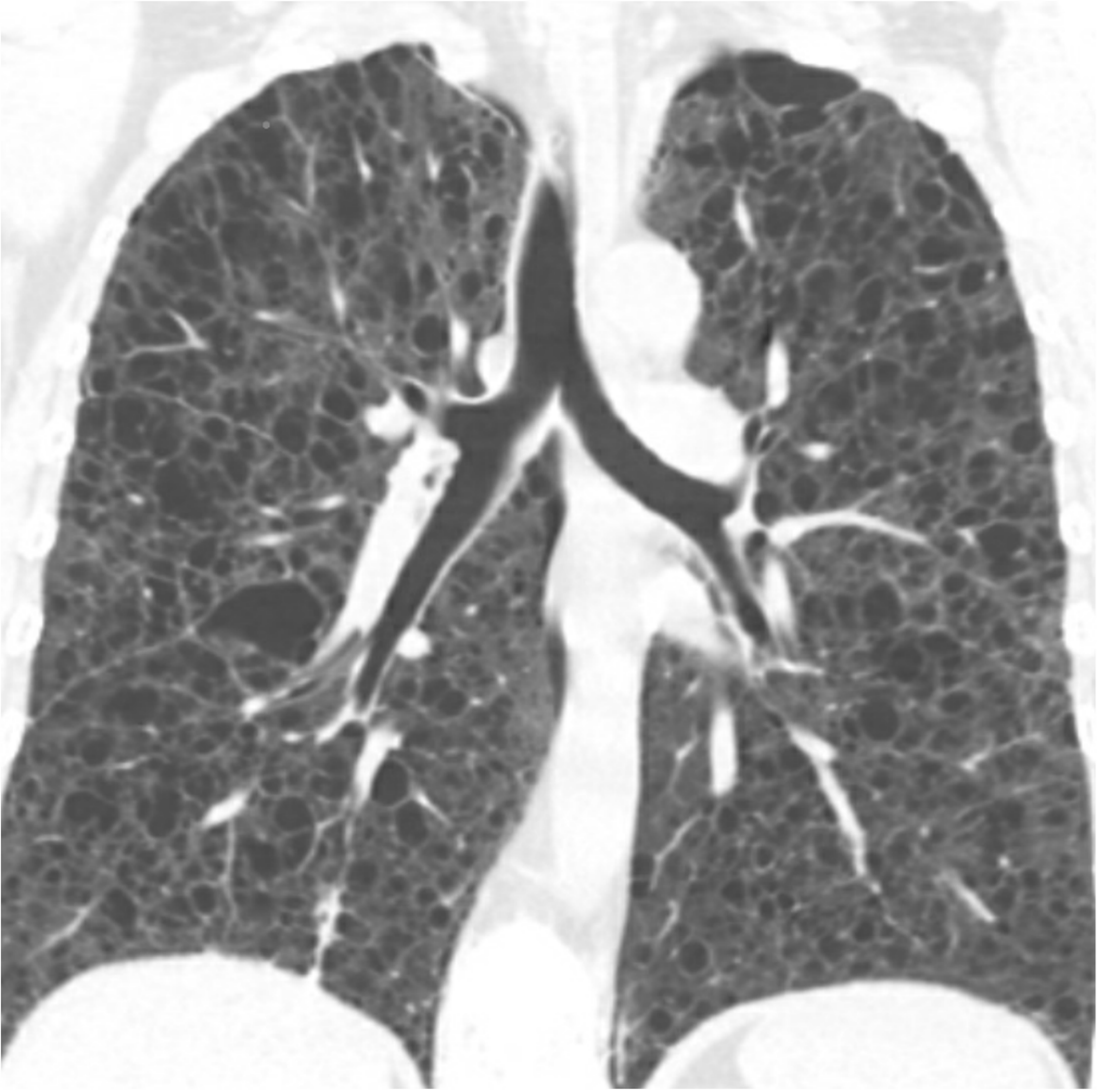
Coronal CT reformatted image of the chest shows diffuse severe pulmonary involvement by thin-walled cysts. Although several cysts are small (2–5 mm), the majority are much larger, measuring up to 12 mm. Note variable cyst shapes including polygonal shapes and a diffuse involvement of the lungs

Despite pleurodesis, she continued to have multiple recurrent, bilateral pneumothoraces. CT chest in the following year showed diffuse cystic changes, with innumerable small, thin wall, well defined cysts seen throughout the entire lung parenchyma bilaterally, ranging in size from 0.2 to 2.8 cm (Fig. 1). The patient was then transferred to our institution for a lung transplant evaluation. She had a bilateral lung transplant (1½ year after the diagnostic biopsy) from a cadaveric donor who was ABO group A+ (recipient A+), cytomegalovirus (CMV) mismatch (donor positive, recipient negative serology). There was no preformed donor specific antigen (DSA) antibody detected. Examination of the explanted lungs confirmed the presence of LAM cells, with involvement of alveolar septa, lymphatics from pleura, interlobular septa, bronchovascular bundles, and peribronchial lymph node sinuses. There was greater than 50% involvement with pulmonary cysts.

After transplantation, subsequent lung allograft biopsies were largely unremarkable. She stated that she was in compliant with transplant medications and did not experience significant side effects. A biopsy at 6 years post-transplant showed mild acute cellular rejection (International Society of Heart and Lung Transplantation [ISHLT] 2007 classification A2 BX). There was also a mild decrease in FEV1 (Forced Expiratory Volume in 1 Second) from 2.7 L to 2.3 L. She was then treated with pulse steroids and later stabilized. Her FEV1 on the next visit increased to 2.5 L.

**Fig. 2:**
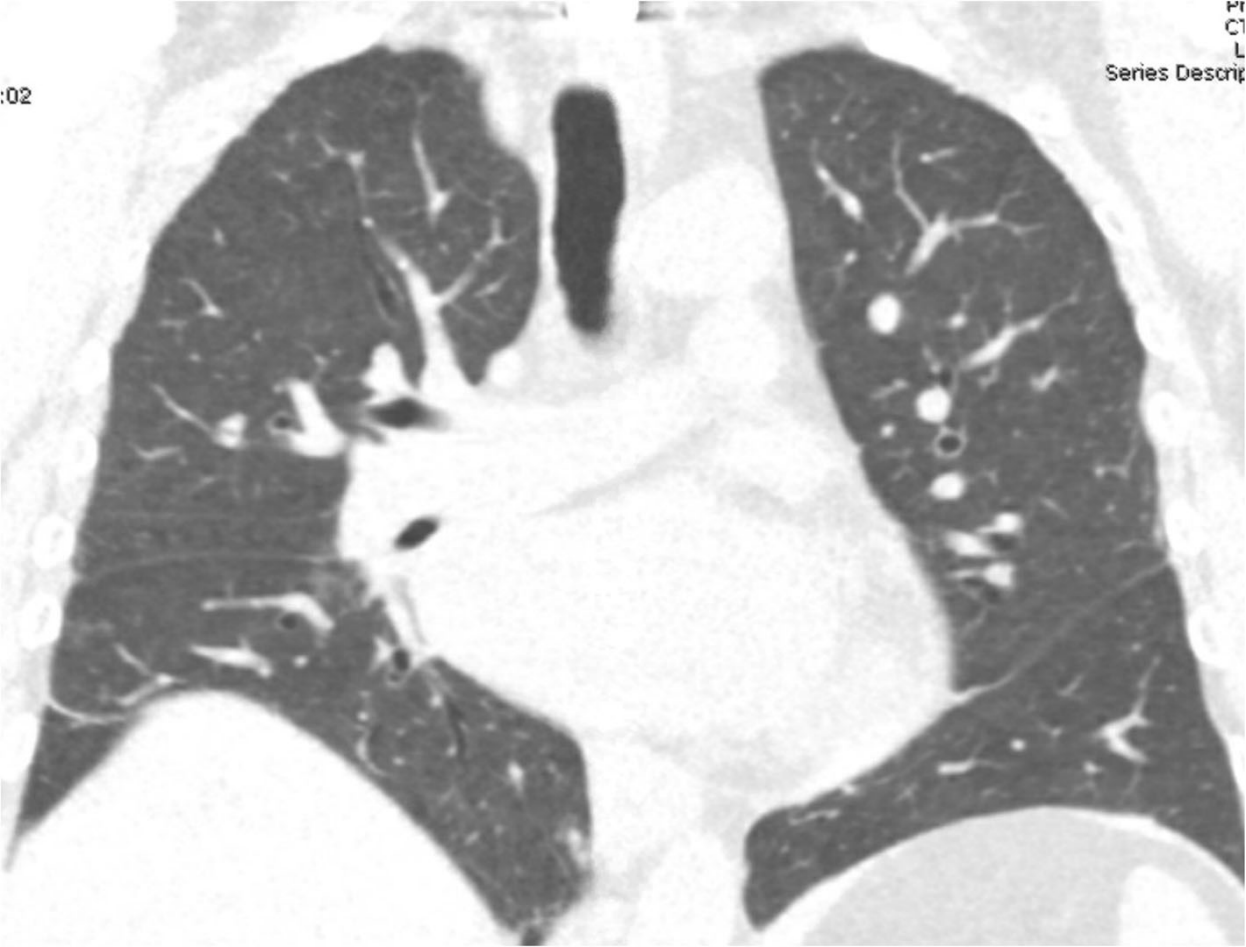
Coronal CT reformatted image of the chest during the admission for the recent viral infection (before Covid) shows clear lungs without any cysts and no evidence of infiltrate

In early 2021, she presented to the emergency department with cough, shortness of breath, and fatigue, which had been ongoing for 1 week. She also had a sore throat 1 day prior to the visit. She reported no fever or chills. Chest X-ray and CT chest revealed no parenchymal opacities or lucencies (Fig. 2). Nasopharyngeal swab for severe acute respiratory syndrome coronavirus 2 (SARS-CoV-2) reverse transcriptase polymerase chain reaction (RT-PCR) was negative. Bronchoscopy with transbronchial biopsy was performed to assess allograft rejection and infection.

## Transbronchial biopsy pathology

**Fig. 3:**
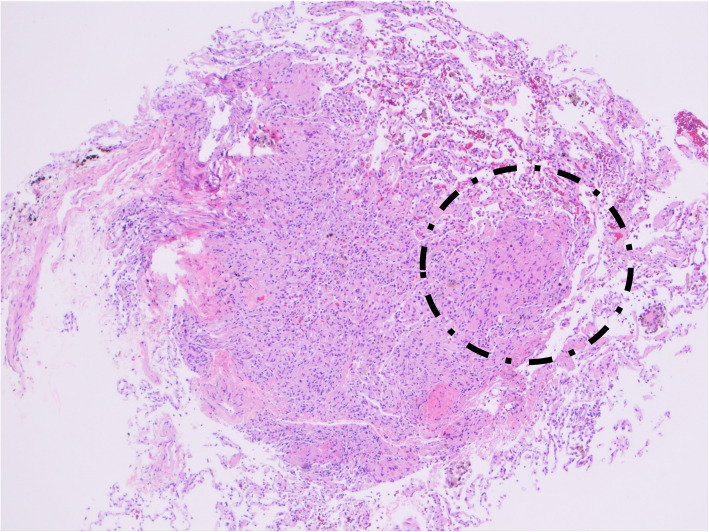
Transbronchial biopsy of the lung allograft shows atypical spindle cell proliferation (dotted circle). The abnormal spindle cells are larger than that of the smooth muscle cells in the nearby arteries and bronchioles (hematoxylin and eosin, original magnification 40X)

**Fig. 4:**
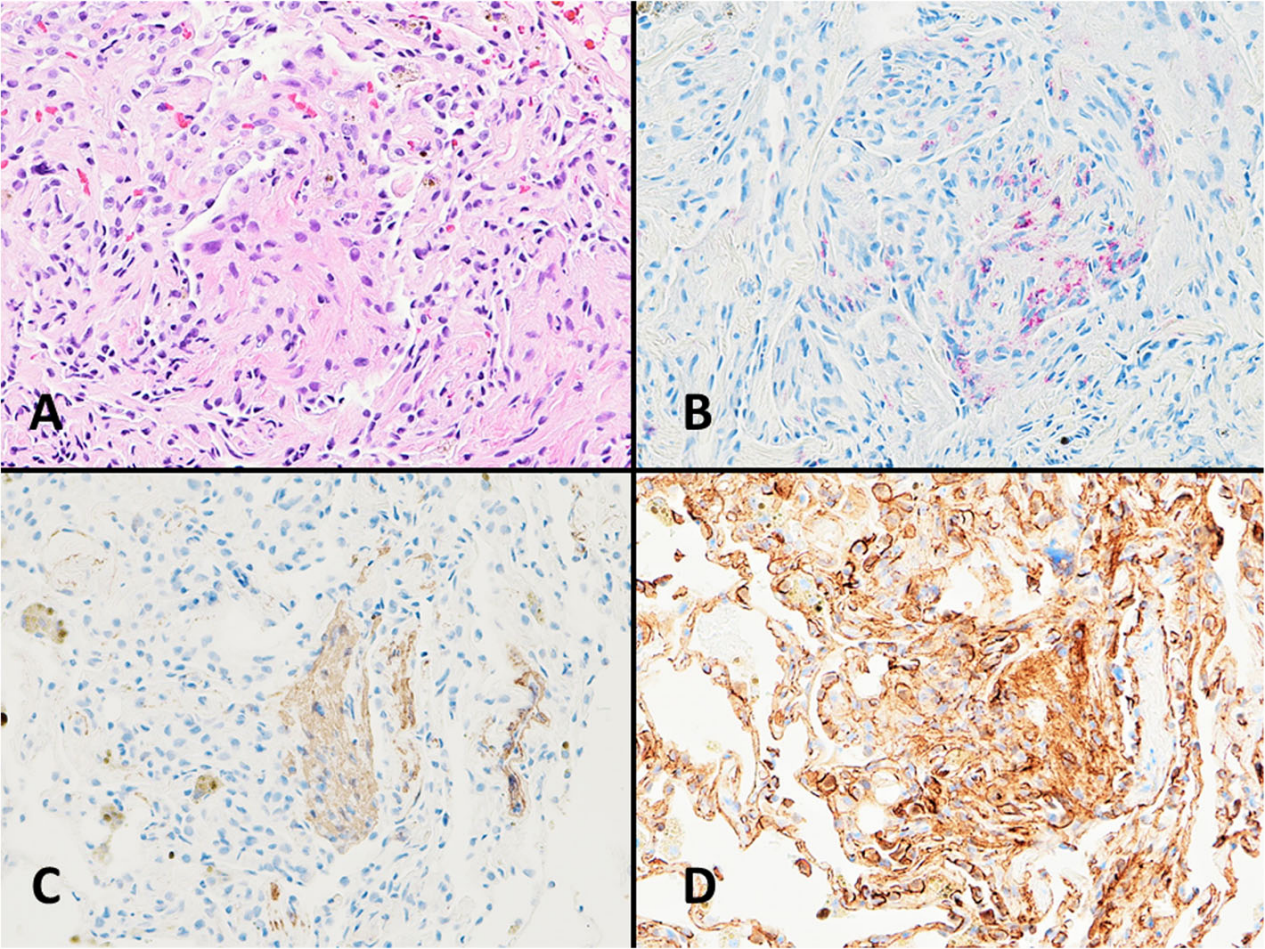
Immunohistochemical studies were performed to categorize the spindle cells. These cells are positive for HMB-45 (**b**), smooth muscle actin (**c**), and show membranous and cytoplasmic staining with beta catenin (**d**). This immunophenotype is consistent with LAM (**a** hematoxylin and eosin, original magnification 200X; **b** HMB-45, original magnification 200X; **c** smooth muscle actin, original magnification 200X; **d** beta catenin, original magnification 200X)

The transbronchial biopsy revealed no evidence of acute cellular rejection or small airway inflammation (ISHLT 2007 classification A0 B0). However, there were foci of spindle cell proliferation, admixed with the bronchovascular bundles (Fig. 3). The abnormal cells were larger in size compare to the surrounding vascular and bronchial smooth muscle cells. Based on the history of LAM in the explanted lungs, immunohistochemical studies were performed. Staining with HMB-45, beta-catenin, and SMA were positive in these cells of interest (Fig. 4). C4d stain was negative in the capillaries. GMS stain was negative for fungus. The overall findings were consistent with recurrent LAM in the lung allograft.

## Progression

Besides SARS-CoV-2 RT-PCR, her nasopharyngeal swab was also submitted for a viral PCR panel, which returned as positive for rhinovirus/enterovirus. She was admitted and received empiric antibiotics and a steroid taper. During the admission, her symptoms gradually improved until she was discharged on the 4th day of hospital stay.

One week after discharge, the patient experienced worsening dyspnea and productive cough. She underwent another nasopaharyngeal swab for SARS-CoV-2 RT-PCR. The result returned as positive. She was then hospitalized with a diagnosis of acute hypoxemic respiratory failure due to COVID-19 pneumonia. Acute respiratory distress syndrome. Over the course of 3 weeks, she was maintained on bilevel positive airway pressure (BIPAP) and received treatment for both possible rejection as well as broad spectrum antibiotics. She suffered acute hemodynamic collapse one evening consistent with a massive bleed, and over the subsequent 24 h developed severe intractable lactic acidosis, did not recover hemodynamic stability, and passed away on day 36 of her hospital stay. Autopsy permission was requested and granted.

## Autopsy findings

**Fig. 5:**
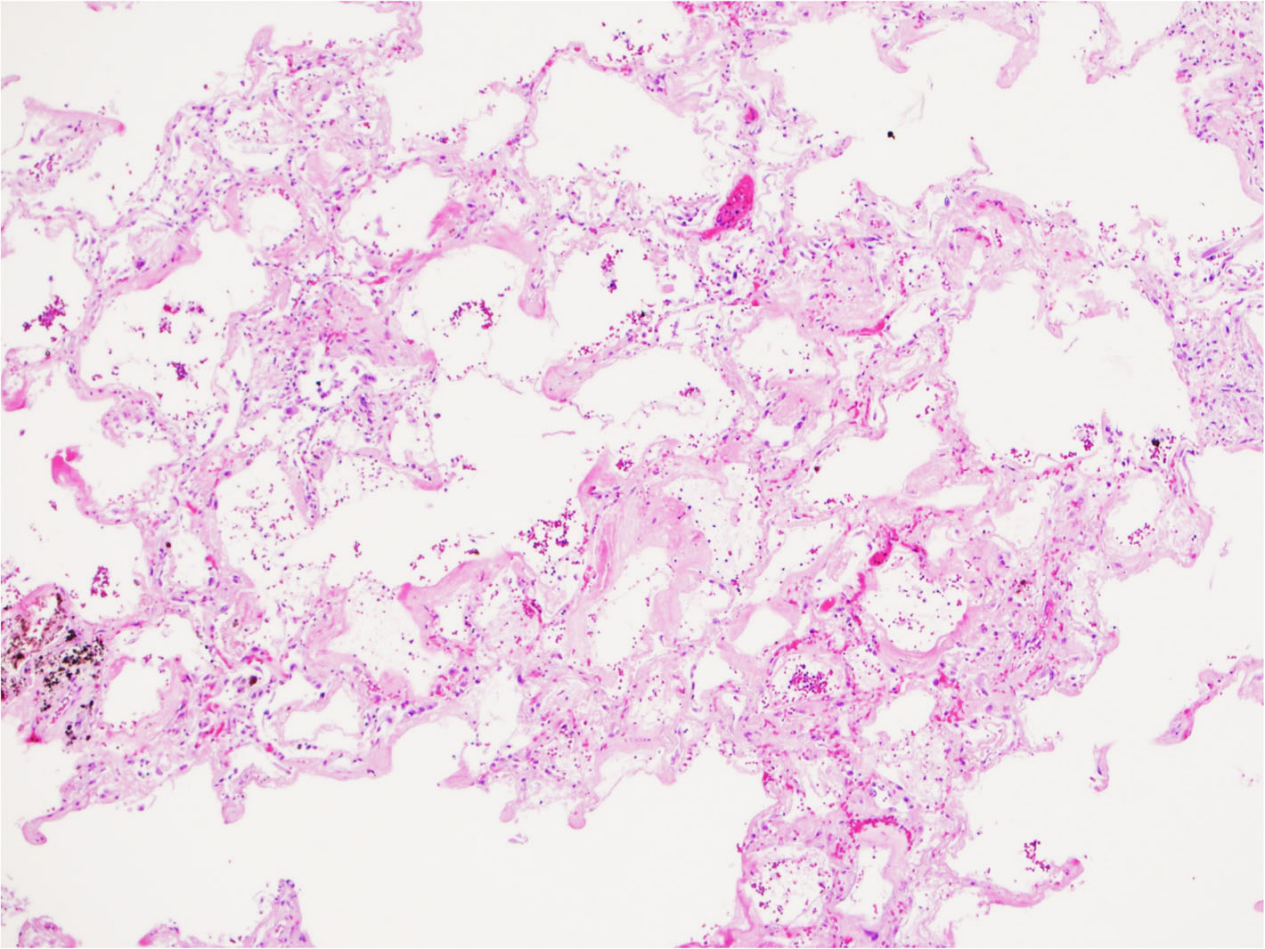
Microscopic examination of the lung from the autopsy shows various stages of diffuse alveolar damage, including hyaline membrane and fibroblast proliferation (hematoxylin and eosin, original magnification 40X)

**Fig. 6:**
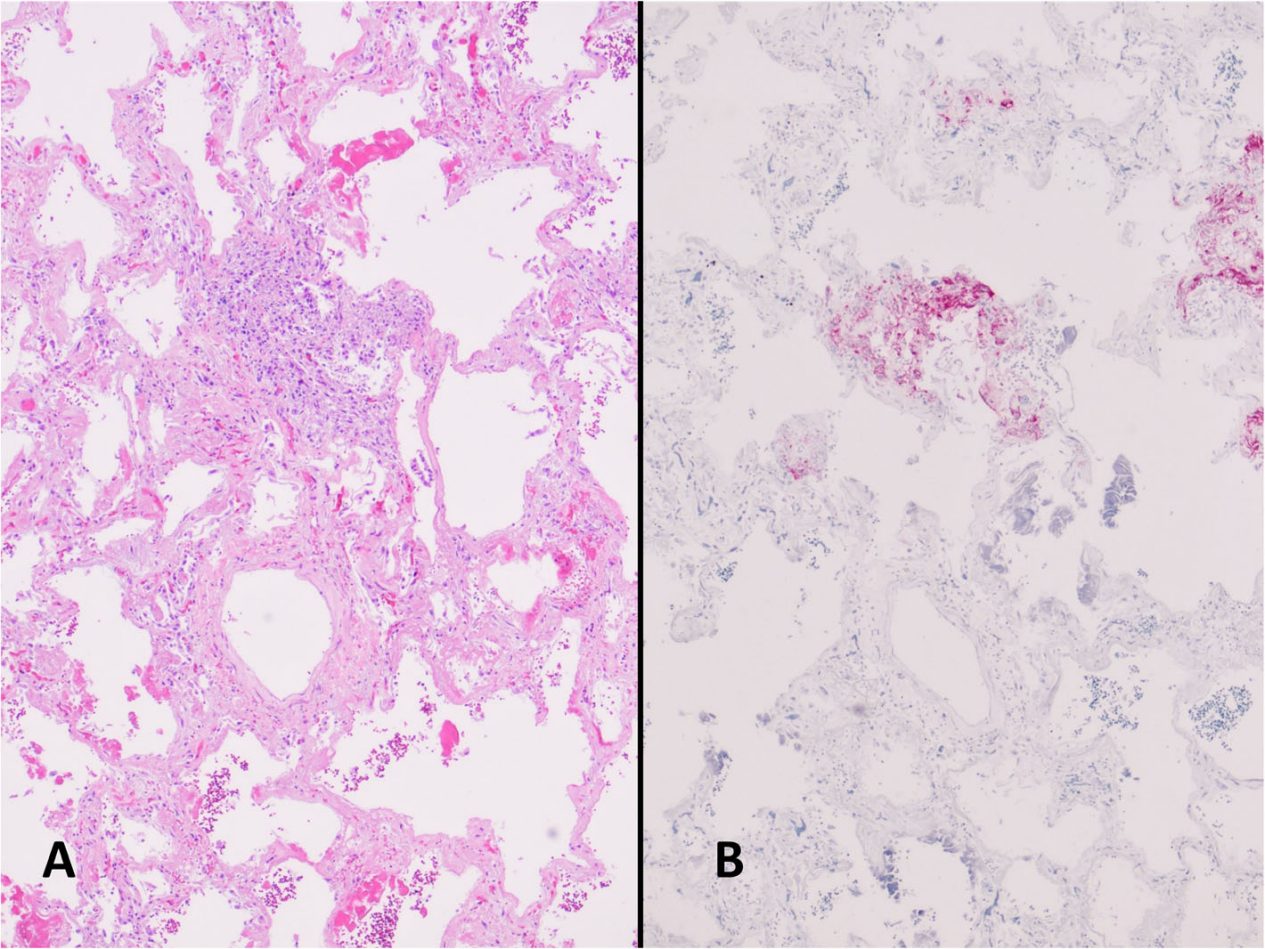
Besides DAD, examination of the lungs confirm the presence of LAM cells (**a**). Immunohistochemical stain for HMB-45 (**b**) confirms the origin of these atypical cells are LAM cells (**a** hematoxylin and eosin, original magnification 40X; **b** HMB-45, original magnification 40X)

On autopsy, salient findings included massive gastrointestinal hemorrhage, with dusky discoloration consistent with mucosal ischemia of the full length of small intestine, and a copious amount of intraluminal frank hemorrhage (2500 mL). No source of bleeding was identified. The lungs were congested, heavy and diffusely consolidated with extensive hepatization of the pleural parenchyma of all lobes (left lung 750 g [expected, 325–480 g]; right lung 840 g [expected, 360–570 g]). There were also significant collections of serosanguinous fluid in the pleural cavities. There was no cystic changes or macroscopic evidence of LAM identified. Microscopic examination of the lungs demonstrated diffuse alveolar damage with acute, organizing and fibrotic stages present (Fig. 5). Multifocal micronodular aggregates of spindled and epithelioid cells surrounding arterioles and within alveolar walls were identified, consistent with recurrent LAM (Fig. 6). HMB-45 stain highlighted these cells. There was also extensive intra-alveolar fluid and mixed neutrophilic and mononuclear cell infiltrates, particularly in the upper lobes. No evidence of arteriosclerosis or pulmonary thromboembolism were present.

Additional autopsy findings included cardiomegaly and coronary atherosclerosis. Lung tissue cultures were positive for vancomycin-resistant *E. faecium*. The cause of death was massive gastrointestinal hemorrhage and sepsis, in a setting of acute respiratory distress syndrome with SARS-CoV-2 (COVID) pneumonia and clinical immunosuppression secondary to lung transplantation.

## Discussion

Lung transplantation in a LAM patient was first reported in 1984 (Estenne et al. 1984). An early international survey in 1996 reported no difference in overall survival rates for transplanted LAM patient to those of emphysema and pulmonary fibrosis, with the overall one-year and two-year survival rates of 69 and 58%, respectively (Boehler et al. 1996). Another case series reported similar survival rates (Pechet et al. 2004). However, later studies after 2000 showed a significant improvement in transplanted LAM patients. Kpodonu et al. (2005) showed significantly favorable survival rates in LAM patient compared to transplant from other causes, with 1-year, 3-year, and 5-year survival rates at 85.75, 76.35, 64.91%, respectively (Kpodonu et al. 2005). A large European case series also revealed the similar overall 1-year and 3-year survival rate of 79 and 73% (Benden et al. 2009). It has been shown by the data from National Heart, Lung, and Blood institute LAM registry study group that transplantation in LAM patients was associated with improvements in pulmonary function and quality of life in comparison to advanced stage LAM patients (Maurer et al. 2007). The latest cohort from Japan revealed the survival rate in transplanted LAM patient to be 86.7% at 1 year, 82.5% at 3 year, 73.7% at 5 year, and 73.7% at 10 year (Ando et al. 2016).

There is little data regarding recurrent LAM in the lung allograft. The recurrence rates in reported case series varied between 6 and 7%.(Ando et al. 2016; Benden et al. 2009; Pechet et al. 2004) Previously reported cases of recurrent LAM in the lung allograft are shown in Table 1. In the 1990’s, LAM in allograft were primarily identified during autopsy (Bittmann et al. 1997; Nine et al. 1994; O'Brien et al. 1995; Pechet et al. 2004; Pigula et al. 1997), but more recent case reports showed that recurrence could be found from transbronchial biopsies for abnormal imaging (Chen et al. 2006; Sugimoto et al. 2008). It can be observed that the majority of the patients died from infection. Only two patients were reported to have LAM as a direct cause of death (Bittmann et al. 1997; Zaki et al. 2016).

**Table 1:** Previously reported cases of recurrent lymphangioleiomyomatosis (LAM) in the lung allograft

Reference	Age at diagnosis	Age at Transplant	Transplant Surgery	Time at recurrence	Means of diagnosis	Outcome
Nine et al. 1994	Unknown	45	Left single lung	3 years	Autopsy	Death at 3 years after Tx from disseminated fungal infection with cerebral abscess
O'Brien et al. 1995	38	42	Right single lung	2 years	Autopsy	Death at 2 years after Tx from invasive pulmonary aspergillosis
Pigula et al. 1997	Unknown	Unknown	Unknown	2 months	Autopsy	Death from herpes pneumonia
	Unknown	Unknown	Unknown	30 months	Autopsy	Death from disseminated aspergillosis
Bittmann et al. 1997, 2003	31	31	Right single lung	442 days	Open lung biopsy	Death several months after the diagnosis of recurrent LAM from respiratory failure
Karbowniczek et al. 2003	Unknown	44	Right single lung	22 months	Autopsy	Aspergillus pneumonia
Pechet et al. 2004	Unknown	Unknown	Right single lung	22 months	Autopsy	Sepsis
Chen et al. 2006, 2009	20	23	Right and left lower lobes	2 years	- Abnormal chest CT - Transbronchial biopsy	Treated with sirolimus for 3 years, alive at report with improved respiratory function and imaging finding
Sugimoto et al. 2008	28	28	Right and left lower lobes	5 years	- Abnormal chest CT - Pleural effusion cytology	Treated with sirolimus for 2 years, alive at report with improved respiratory function
Zaki et al. 2016	51	66	Bilateral lungs	9 years	Transbronchial biopsy	- Sirolimus therapy stabilized lung function for a brief period - Subsequently developed H1N1 influenza and mycoplasma pneumonia - Death from chronic rejection and recurrent LAM
Current case, Sathirareuangchai et al. 2021	44	45	Bilateral lungs	7 years	Transbronchial biopsy	- Death from COVID-19

Matsui et al. published a LAM histologic grading system in 2001, namely Lymphangioleiomyomatosis Histologic Score (LHS). The grading scheme is based on the percentage of tissue involvement by the cysts and infiltration by LAM cells (LHS-1, < 25%; LHS-2, 25–50%; LHS-3, > 50%). Based on the explant pathology, the LAM histologic grade in the current case corresponded with LHS-3. According to Matsui et al., LHS-3 has the worst overall survival with the 5- and 10-year survival of 62.8 and 52.4%. There was no available data on recurrent disease in their series. Since the number of recurrent cases in LAM is still low, there is no reported data on how recurrence actually affects the outcome of the transplant.

The pathogenesis of recurrence was first purposed that the LAM cells were derived from the donor cells, based on a positive result of in situ hybridization for the Y chromosome (Nine et al. 1994). A later case report also supported this theory by demonstrating in situ hybridization of the Y chromosome (Bittmann et al. 2003). However, in a more recent study using microsatellite analysis and TSC2 gene mutational analysis, it was proved that LAM cells were actually of recipient origin (Karbowniczek et al. 2003). The data support the theory about LAM pathogenesis in the native lung, wherein LAM cells originate from the smooth muscle component of renal angiomyolipoma or other sites rather than a primary disease of the lung (Karbowniczek et al. 2003).

Sirolimus has been used in the treatment of LAM after 2000s. It has been shown to effectively stop the decline in lung function and stabilize the disease (McCormack et al. 2011). It could be one of the reasons why the transplanted LAM patients tend to have better outcome. Few case reports demonstrated that sirolimus may be beneficial in patients with recurrent LAM (Chen et al. 2009; Sugimoto et al. 2008). In the current case, the patient was transferred from an outside facility to our institution for lung transplant, therefore it is unknown for us why she was not on sirolimus. After transplant, she continued to be asymptomatic with unremarkable chest imaging. Sirolimus was considered when the most recent biopsy revealed recurrent LAM. Unfortunately, her diagnosis of severe COVID-19 postpone the decision to initiate sirolimus.

Immunosuppressive status in lung transplant patient poses the higher risk to COVID-19. Two large case series of lung transplant recipients with COVID-19 revealed that the majority of patients (84–88.6%) required either hospitalization or respiratory support, with the mortality rate of 14.3–39% (Messika et al. 2021; Saez-Gimenez et al. 2021). Risk factors associated with mortality include obesity (Messika et al. 2021), worse respiratory status and CXR on admission, higher serum D-dimer, interleukin-6, and lactate dehydrogenase (Saez-Gimenez et al. 2021). The pathology findings of COVID-19 in this case resembled those previously reported, mainly diffuse alveolar damage (Borczuk et al. 2020; Konopka et al. 2020). However, there was no evidence of either thromboembolism or fibrin thrombi in the current case.

## Conclusion

Recurrent LAM in the lung allograft can be easily overlooked in the surveillance transbronchial biopsy. Although its significance in asymptomatic patients is yet to be determined, pathologists should be aware of the explant diagnosis and carefully screen for atypical smooth muscle-like cells in allograft surveillance biopsies. Immunohistochemical studies are useful in identification of these tumor cells.

## Data Availability

Data sharing is not applicable to this article.

## Abbreviations

LAM: Lymphangioleiomyomatosis
SMA: Smooth muscle actin
LHS: Lymphangioleiomyomatosis Histologic Score
ISHLT: International Society of Heart and Lung Transplantation
SARS-CoV-2: Severe acute respiratory syndrome coronavirus 2
COVID-19: Coronavirus disease
